# Novelties in *Uvaria* (Annonaceae) from West Africa

**DOI:** 10.3897/phytokeys.271.188903

**Published:** 2026-03-10

**Authors:** Carel C. H. Jongkind

**Affiliations:** 1 Botanic Garden Meise, Nieuwelaan 38, B-1860 Meise, Belgium Botanic Garden Meise Meise Belgium

**Keywords:** Annonaceae, IUCN Red List, Liberia, new species, taxonomy, *
Uvaria
*, West Africa

## Abstract

A new species of *Uvaria* (Annonaceae) from Liberia, only known from one fruiting collection, is described and illustrated. *Uvaria tobliensis***sp. nov**. differs from all other African *Uvaria* species by the combination of a relatively small leaf blade (4–6.5 × 1.8–2.8 cm) with a very dense indumentum on the lower surface, and relatively large subglobose monocarps (2.2–2.5 × 1.9–2.1 cm). The conservation status of the new species is assessed as CR following the IUCN Red List Categories and Criteria. The two subspecies of *Uvaria* ovata, subsp. *ovata* and subsp. *afzeliana*, are treated here as distinct species, *U. ovata* and *U. leonensis*, and lectotypes are designated.

## Introduction

*Uvaria* L. (Annonaceae) is a genus of around 200 species of lianas or scrambling shrubs from the Old World tropics (Couvreur et al. 2022). In West Africa *Uvaria* species are easy to recognise by the combination of their special climbing habit and the presence of fasciculate or stellate hairs.

**Figure 1.**
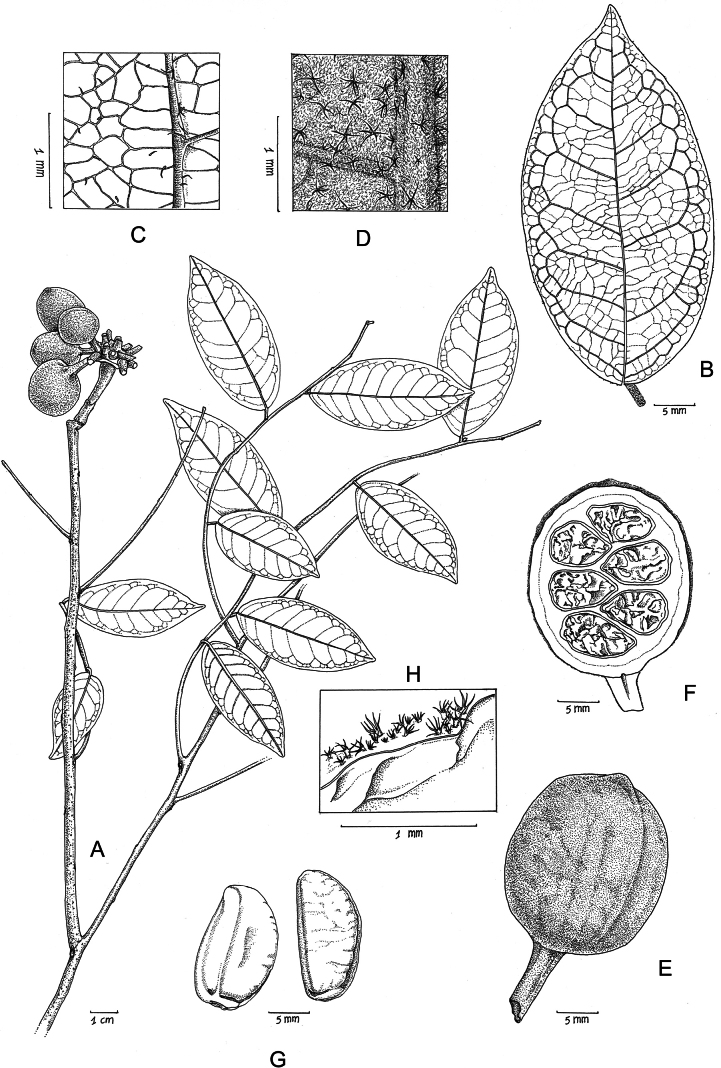
*Uvaria tobliensis*. **A**. Branch with fruits; **B**. Leaf from above; **C**. Leaf above, detail; **D**. Leaf below, detail; **E**. Monocarp; **F**. Monocarp, longitudinal section; **G**. Seeds; **H**. Monocarp, detail of indumentum. Drawing by Hilde Orye from *JWA Jansen 874* (monocarp, including seeds, from spirit).

One of the 17 *Uvaria* species included in the keys in the “Woody plants of Western African forests” is still undescribed (*Uvaria* sp. A, Hawthorne and Jongkind 2006: 56, 57). The only specimen of this species is in fruit, the flowers are still unknown, but its characters are such that it can’t be confused with any other African *Uvaria*. With the Annonaceae issue of the Flora of Cameroon, published after 2006, it can also not be identified (Couvreur et al. 2022). It can be recognised by its relatively small leaves with a very dense indumentum on the lower side and the shape and size of its monocarps. It is named here *U. tobliensis* Jongkind, sp. nov. (Fig. 1). In 2024, the author visited the area north-east of Tobli/Toetown in Liberia, where it was collected in 1968, without finding this species but noticing the strong pressure on what is left of the forest.

**Figure 2.**
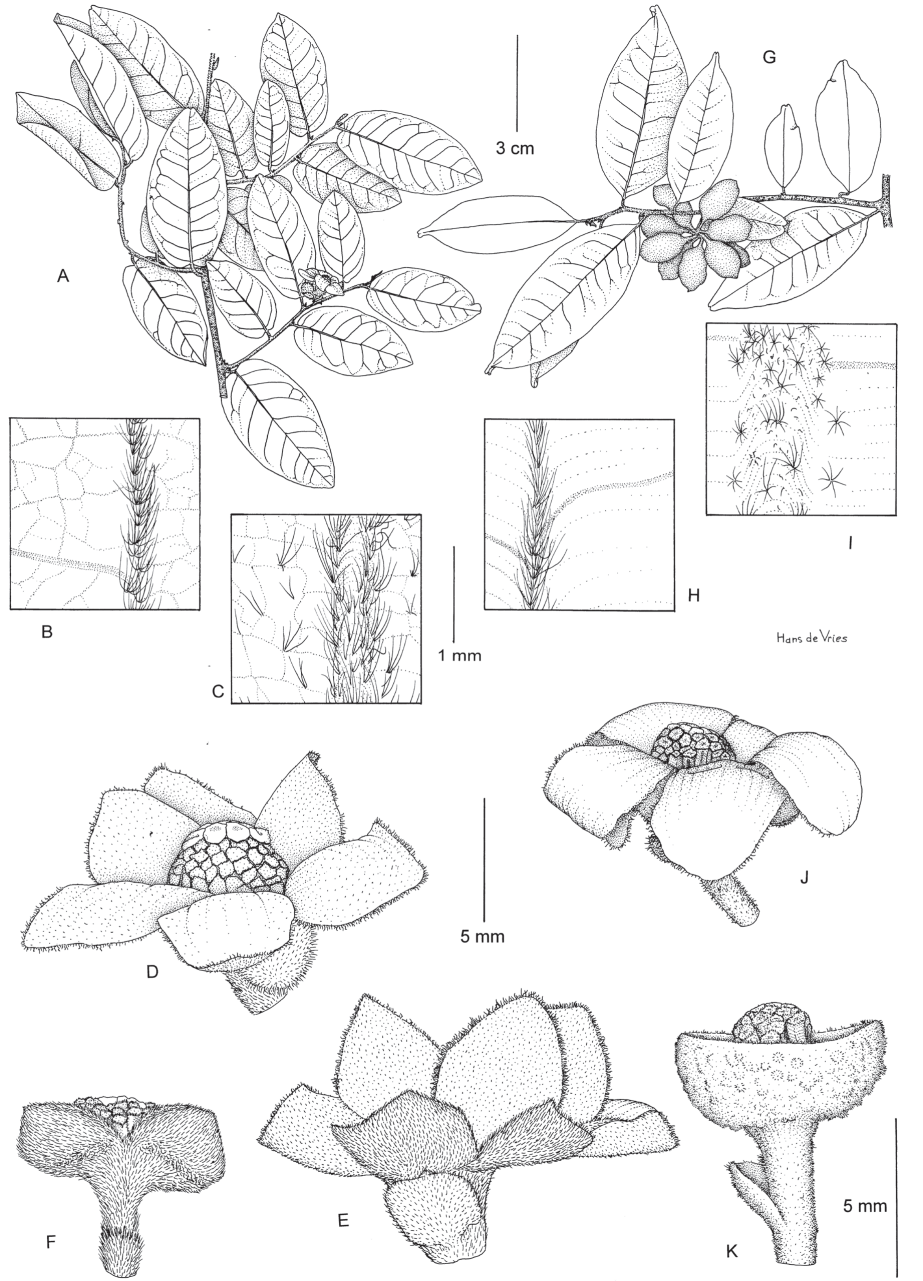
*Uvaria ovata*. **A**. Plant with inflorescence; **B**. Detail of leaf above, with midrib; **C**. Detail of leaf beneath, with midrib; **D**. Flower from above; **E**. Flower from aside, showing calyx; **F**. Flower without petals showing torn calyx cup. *Uvaria leonensis*. **G**. Plant with infructescence; **H**. Detail of leaf above, with midrib; **I**. Detail of leaf beneath, with midrib; **J**. Flower; **K**. Flower without petals, showing entire calyx cup. **A, D, E** from *Bokdam 2779*; **B, C, F** from *Berg & Wiebes 1442*; **G** from *Jongkind 10707*; **H–K** from *Jongkind 11372*. Drawing by Hans de Vries.

More than seventy years ago Keay studied the West African *Uvaria* species for the second edition of the “Flora of West Tropical Africa” (Keay 1954). As a result of his study, he lumped under *U. ovata* (DC.) A.DC. several species that were still recognised in the first edition (Hutchinson and Dalziel 1927), and separated two subspecies, subsp. *ovata* and subsp. *afzeliana* (DC.) Keay. These subspecies were recognised by him by differences in the leaves (Keay 1952) and he writes that both have the same flowers and fruits. More specimens of these two taxa have been collected since, and revealed that, apart from the leaf characters, there are important differences in the flowers and fruits as well (Fig. 2, Table 1). The two taxa are different enough to be recognised as species and are treated here as such.

**Table 1.** Principal differences between *U. ovata* and *U. leonensis*.

	*Uvaria ovata*	*U. leonensis*
Midrib of the leaf beneath	Dense appressed stellate-fasciculate hairy	Scattered stellate-fasciculate hairs with spreading branches
Leaf base	Cordate to rounded	Obtuse to cuneate
Calyx shape (open flower)	Deeply split	Cupular with entire margin
Calyx indumentum	dense appressed stellate-fasciculate indumentum outside	short velutinous stellate-fasciculate indumentum outside
Fruit shape	Monocarp subglobose, up to 1.5 × as long as wide	Monocarp almost cylindrical, often 2 × as long as wide
Fruit indumentum	covered with dense small, spreading stellate-fasciculate hairs (velutinous)	covered with dense small, appressed stellate-fasciculate hairs (not velutinous)

20 years ago we counted in “western Africa” or “Upper Guinea” (in this case the countries west of Benin) 17 *Uvaria* species (Hawthorne and Jongkind 2006). With two lumped in the time since 2006 (*U. platyphylla* Boutique & *U. anonoides* Baker f.) and two species added here, we have now 18 *Uvaria* species. However, there are still *Uvaria* specimens from western Africa that can’t be placed and might represent undescribed species.

## Material and methods

This paper is based on a study of herbarium collections in BM, BR, K, P, and WAG, as well as on field studies by the author in Liberia, Guinea, Ivory Coast and Ghana. The Tropicos website from Missouri Botanical Garden [http://www.tropicos.org], the JSTOR Global Plants website [https://plants.jstor.org/] and the Global Biodiversity Information Facility [https://www.gbif.org/] were used to find images of herbarium specimens from additional herbaria. The conservation status of the new species was assessed following the Guidelines for Using the IUCN Red List Categories and Criteria (IUCN 2024).

## Taxonomic treatment

### Uvaria tobliensis

Taxon classificationPlantaeMagnolialesAnnonaceae

Jongkind sp. nov.

E77451A6-799F-5CC5-9548-5CC5D58E3A21

urn:lsid:ipni.org:names:77377627-1

Figs 1, 3

Uvaria sp. A (Hawthorne and Jongkind 2006: 57).

#### Type material.

Liberia • **Grand Gedeh**, “along the road from Tapita to Chien, 13 miles NE of Tobli” (see Note), 17 Jul 1968, fr., *JWA Jansen 874* (***holotype***: WAG [WAG.1418465]; ***isotype***: U [U.1074856]).

**Figure 3.**
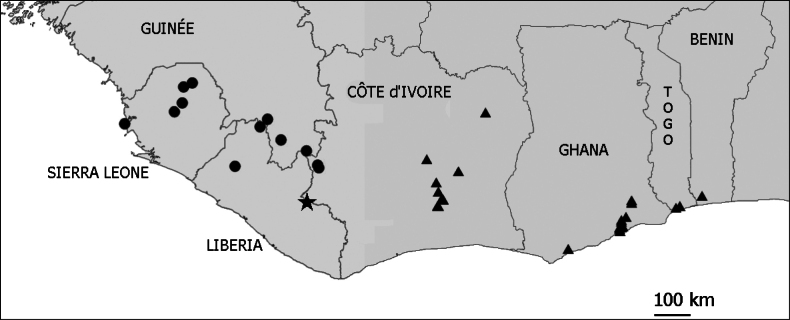
Distribution map of *Uvaria leonensis* (dots), *U. ovata* (triangles) and *U. tobliensis* (star).

#### Diagnosis.

Differs from all other African *Uvaria* species by the combination of a relatively small leaf blade (4–6.5 × 1.8–2.8 cm) with a very dense, mostly stellate-fasciculate, indumentum on the lower surface, and relatively large subglobose monocarps (2.2–2.5 × 1.9–2.1 cm).

#### Description.

***Liana***. Young ***shoots*** with short brownish indumentum of, at least partly, stellate-fasciculate hairs, soon with many small lenticels. ***Petiole*** 2–3 mm long, with short brown indumentum. ***Leaf blade*** elliptic, 4–6.5 × 1.8–2.8 cm, covered beneath with a dense layer of appressed, small, pale green hairs, mostly stellate-fasciculate, mixed with scattered larger (but still small) stellate-fasciculate hairs, above almost glabrous except for the midrib, dark green, base rounded, apex acuminate; midrib impressed above; 7–9 pairs of secondary veins; tertiary venation reticulate. ***Monocarps*** stipitate, stipes slightly excentric, 10–13 mm long, 3–4 mm in diameter; monocarps c. 15, subglobose, with only a faint ridge along one side, 2.2–2.5 × 1.9–2.1 cm, brown, with dense short, mostly stellate-fasciculate, erect hairs, with 5–7 seeds in two rows. ***Seeds*** 14–16 × 8–10 × 5–6 mm, roughly triangular in cross-section, smooth, endosperm ruminate.

#### Etymology.

The new species is named after the village near the type location.

#### Distribution and ecology.

Only known from the type location in high forest in Liberia (Fig. 3).

#### Conservation status.

The only specimen of *U. tobliensis* is collected in Liberia, most likely along the c. 8 km long road between Tobli and the border with Côte d’Ivoire. There are still large patches of forest along this road but most of the forest is converted to farmland. There is no protected forest anywhere nearby. Following the Guidelines for Using the IUCN Red List Categories and Criteria (IUCN 2024) it is very hard to assess this species as anything else other than Critically Endangered (CR, according to criterion B). The EOO cannot be calculated and the AOO is 4 km^2^.

#### Note.

The location on the herbarium sheet, “along the road from Tapita to Chien, 13 miles NE of Tobli”, can’t be correct. The main road from Tapita, where Jansen started, to Chien (Zwedru) goes roughly north-west to south-east, never to the north-east. The road that branches from the Tapita-Zwedru road at Tobli and that goes north-east is the main road to Côte d’Ivoire. However, long before you have travelled 13 miles (c. 21 km) on this almost straight road you reach the Ivorian border. The collecting location of the type is most likely somewhere along this c. 8 km long road between Tobli and the Ivorian border. In the field notebook of JWA Jansen the collecting location for that day is just described as “Woodcuttlery of Mr Charmois” but that could not be located anymore.

### Uvaria leonensis

Taxon classificationPlantaeMagnolialesAnnonaceae

Engl. & Diels, Notizbl. Königl. Bot. Gart. Berlin 2: 293 (1899)

BE338DCE-81FC-58F1-9A7B-E976F821102D

Figs 2G–K, 3, 4

= Uvaria ovata (DC.) A.DC. subsp. *afzeliana* (DC.) Keay (Keay 1952: 544; 1954: 35). Type material. Sierra Leone • s.l., 1792–1794, *Afzelius* s.n. (holotype: B destroyed; lectotype, designated here: BM [BM000554057]).
≡ Unona ovata DC. var. *afzeliana* DC., Syst. Nat. 1: 489 (1817). Type material. Sierra Leone • s.l., 1792–1794, *Afzelius* s.n. (holotype: B destroyed; lectotype, designated here: BM [BM000554057]).
= Uvaria nigrescens Engl. & Diels, Monogr. Afrik. Pflanzen.-Fam. 6: 15 (1901). Type material. Sierra Leone • s.l., 1792–1796, *Afzelius* s.n. (holotype: B [B100153102]).

#### Distribution and ecology.

*U. leonensis* is known from several locations roughly between the Danané area in western Côte d’Ivoire and Freetown in Sierra Leone (Fig. 3) between (100 ? -) 250–600 m altitude. This is an area with a high rainfall combined with a strong dry season. *U. leonensis* is found in forest borders and thickets, it seems to prefer rocky soils.

**Figure 4.**
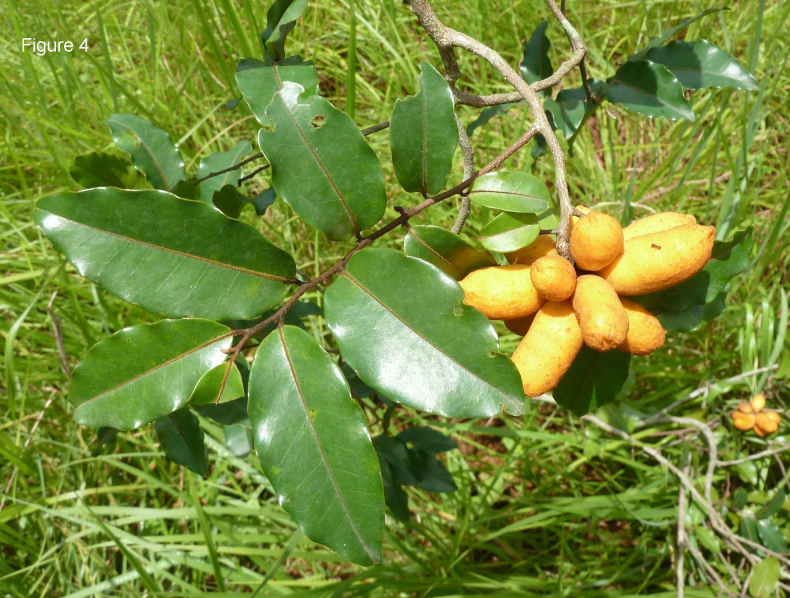
*Uvaria leonensis*. Fruiting branch. From *Jongkind et al. 11372* by Carel Jongkind.

#### Lectotypifications.

The isotype of *Unona ovata* var. *afzeliana* and *Uvaria leonensis* in BM is selected here as lectotype for the two taxa to replace the destroyed holotype. This sheet was annotated as isotype of *Unona ovata* var. *afzeliana* by Keay and he explains that both names have the same type (Keay 1952).

#### Notes.

*Uvaria nigrescens* Engl. & Diels was made synonym of *U. chamae* P.Beauv. by Keay (Keay 1952). He probably did not see a type specimen but made this decision on the basis of the description and illustration published by Engler and Diels (1901). The holotype of *U. nigrescens* in Berlin is almost identical to the type of *U. leonensis* and the name is here transferred to that species as a new synonym. The leaves, flowers and fruits of *U. chamae* are clearly larger than those of *U. leonensis* and its leaf base is usually rounded, not obtuse to cuneate. Both *U. nigrescens* and *U. leonensis* are based on Afzelius specimens from Sierra Leone, but the first on a fruiting specimen and the second on a flowering specimen.

#### Selection of specimens seen.

Côte d’Ivoire • Sommet du Mont Goula, près Danané, 10 Apr 1909, fr., *Chevalier* 21210 (BR, P); • 2 km E of Danané, 6 Mar 1959, fl., *Leeuwenberg* 2994 (BR, K, MO image, WAG).

Guinea • Mont Djiba, 28 Jul 1949, fr., *Adam 5850* (MO image, P); • Forêt Classée de Mt Yonon, 10 May 2011, fl., fr., *Jongkind* 10707 (BR, G, K, MA, MO, P, WAG); • West of the Nimba mountains, 3 Jul 2012, fl., fr., *Jongkind* 11372 (K, MO, P, WAG).

Liberia • near Soplima, 1 Nov 1947, fr., *Baldwin* 10053 (K, MO image); • Kpelle Forest, south-east of Gainkpa, 18 Dec 2010, ster., *Jongkind* 10249 (WAG).

Sierra Leone • Sula Mountains, Tonkolili River near former village Farangbaya, 29 Jul 2013, fr., *Momoh 17* (BR, MO image, WAG); • Yiben Dam area, Seli River, 4 Aug 2016, fr., *Momoh 140* (WAG); • on rocks near top of Sugar Loaf, 26 Apr 1892, fl., *Scott Elliot 5774* (K); • Rowala, 23 Jul 1914, fr., *NW Thomas 1056* (K).

### Uvaria ovata

Taxon classificationPlantaeMagnolialesAnnonaceae

(DC.) A.DC., Mém. Soc. Phys. Genève 5: 205 (1832)

FFE7E961-226A-5ABC-ACCE-0C80ADA3B33B

Figs 2A–F, 3, 5

≡ Uvaria ovata (DC.) A.DC. subsp. *ovata* (Keay 1952: 544; 1954: 35).
≡ Unona ovata DC., Syst. Nat. [Candolle] 1: 489 (1817). Type material. Ghana • s.l., 1799–1803, *Thonning* s.n. (lectotype, designated here: C [C10004672]; isolectotype: C [C10004673]).
= Uvaria cordata Schumach. & Thonn., Beskr. Guin. Pl. 255 (1827). Type material. Ghana • s.l., 1799–1803, Thonning s.n. (lectotype, designated here: C [C10004672]; isolectotype: C [C10004673]).
= Uvaria globosa Hook.f., Niger Fl. [W. J. Hooker]. 210. (1849). Type material. Ghana • Accra, s.d., *Vogel* s.n. (lectotype, designated here: K [K00198789]; isolectotype: K [K000198788]).
= Uvaria globosa Hook.f. var. *warneckei* Engl., Monogr. Afrik. Pflanzen.-Fam. 6: 57, 1901. Type material. Togo • prope Lome, May 1900, fl., *Warnecke 147*; holotype: B [B 10 0153107] image; isotypes: GOET [GOET005690] image, HBG [HBG502493, HBG502494] images, K [K000041232, K000041233, K000041234], L [L.1770697], M [M0107941] image).

#### Distribution and ecology.

*U. ovata* is found in two disjunct areas (Fig. 3), one area in central Côte d’Ivoire and one in the south of the Dahomey Gap from Ghana to Benin (Hall and Swaine 1981, Akoègninou et al. 2006). Both areas have more or less the same kind of vegetation and climate. *U. ovata* grows in forest borders and thickets between 10–270 m above sea level.

**Figure 5.**
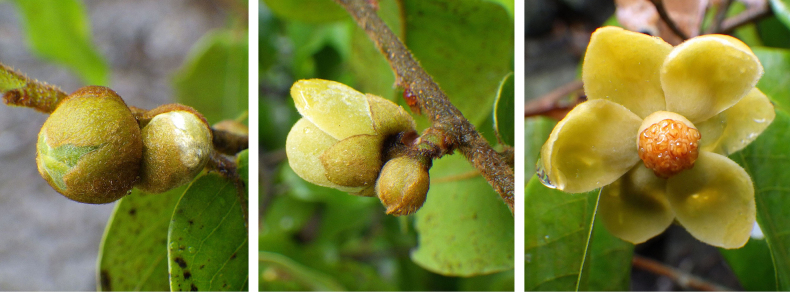
*Uvaria ovata*. From flower bud to open flower. The, at the start, completely closed calyx cup is gradually tearing into three lobes. With one completely closed bud visible in the middle. From *Lachenaud et al. 2762* by Olivier Lachenaud.

There are two recent collections from this species from disturbed vegetation at the north end of São Tomé, both from almost the same location: *Lachenaud et al. 2762* in flower (photos seen) and *Eduardo et all. 94* in fruit (COI image). An older specimen, from March 1968, is cited in a checklist of the island (Figueiredo et al. 2011). No older collections from São Tomé are known. There is a distance of more than 800 km between this location and the population on the mainland. More research is needed to confirm that it is not a relatively recent introduction.

#### Lectotypifications.

*Unona ovata* and *Uvaria cordata* have the same type in Copenhagen (Keay 1952), one of the two sheets is selected here as lectotype. One of the two sheets mentioned by Keay as holotype of *U. globosa* in Kew is also selected here as lectotype of this name.

#### Selection of specimens seen.

Côte d’Ivoire • Réserve de Bouna, près de Kakpin, 20 Jun 1968, fr., *Aké Assi 10297* (UCJ image); Abli-Aloukro, 2 May 1971, *Audru 3927* (P, WAG); Koffie-Yaboué, 17 May 1971, fl., *Audru 4172* (P); Lomo Nord, 10 Oct. 1971, fr., *Audru 4353* (P); Lamto area, 12 Nov 1982, fl., *Berg & Wiebes 1442* (WAG); Orumbo Boka, 12 Jun 1968, fl., fr., *Bokdam* 2779 (BR, MO image, WAG); Assakra, 3 Oct 1956, *Mangenot 4119* (UCJ image); Lamto, Chemin à Zagoussi, 12 Jun 1971, fl., fr., *Miège* s.n. (WAG); route de Morénou (Rocher), fl., Oct 1895, *Pobéguin 203* (P).

Ghana • between Akwamu West and Ajena, 2 Jun 1957, fl., fr., *Adams 4796* (K, WAG); Legon Botanical Gardens, 1 Jul 2010, *van Andel et al. 5646* (WAG); Achimota, 2 May 1974, fl., *Enti FE 1284* (BR, WAG), Achimota Forest, 1 Nov 1993, fr., *Jongkind & Noyes 1278* (BR, K, MO, WAG); ca 20 mls E of Sekondi, 1 Apr 1954, *Morton A 485* (K); on top of Aburi scarp, 4 Jun 1953, fl., fr., *Morton A 1005* (K); 3 mls above Ajena, 29 Nov 1953, fr., *Morton GC 9453* (K).

Benin • Ouèdèmè-Péda, 22 Feb 1999, fl.bud, *Essou* 1539 (WAG).

São Tomé and Príncipe • São Tomé, Côte Nord, ± 0.75 km à l’est de Lagoa Azul vers Morro Peixe, 28 Oct 2019, fl., *Lachenaud et al. 2762* (BR, COI image).

## Supplementary Material

XML Treatment for Uvaria tobliensis

XML Treatment for Uvaria leonensis

XML Treatment for Uvaria ovata

## Additional information

### Use of AI

No use of AI was reported.

### Author ORCIDs

Carel C. H. Jongkind https://orcid.org/0000-0002-1491-2030

### Data availability

All of the data that support the findings of this study are available in the main text.
